# Life‐threatening anaphylaxis in children with cow's milk allergy during oral immunotherapy and after treatment failure

**DOI:** 10.1002/iid3.607

**Published:** 2022-03-29

**Authors:** Laura Badina, Francesca Burlo, Beatrice Belluzzi, Sara Babich, Irene Berti, Egidio Barbi

**Affiliations:** ^1^ Department of Pediatric Institute for Maternal and Child Health, IRCCS Burlo Garofolo Trieste Italy; ^2^ Department of Medicine, Surgery, and Health Sciences University of Trieste Trieste Italy

**Keywords:** allergy, anaphylaxis, children, cow milk, immunotherapy

## Abstract

**Background:**

Oral immunotherapy (OIT) is a promising therapeutic approach for children with persistent IgE‐mediated cow's milk allergy (CMA) but data are still limited.

**Objective:**

To analyze the prevalence of life‐threatening anaphylaxis in children with persistent CMA undergoing OIT and to evaluate potential risk factors.

**Methods:**

This is a retrospective cohort study among children with persistent CMA undergoing OIT over a 20‐year period, following a specific Oral Tolerance Induction protocol. Adverse reactions during the whole period and data on long‐term outcome were registered. Descriptive and nondescriptive statistics were used to describe data.

**Results:**

Three hundred forty‐two children were evaluated. During OIT, 12 children (3.5%) presented severe anaphylactic reactions that needed an adrenaline injection. None required intubation, intensive care unit (ICU) admission, or showed a fatal outcome. Five of them abandoned OIT, five reached unrestricted diet and the others are still undergoing OIT. As far as outcome is concerned, 51.2% reached an unrestricted diet; 13.5% are at the build‐up stage; and 28.0% (97 patients) stopped the OIT. Among these 96 children, 6.3% experienced a severe reaction induced by accidental ingestion of milk with two fatal outcomes.

**Conclusions:**

The risk of life‐threatening reactions was nearly two times lower (3.5% vs. 6.3%) among patients assuming milk during OIT than in those who stopped the protocol. A trend in favor of more severe reactions, requiring ICU admission, or fatal, was shown in patients who stopped OIT.

## Graphical abstract

Oral immunotherapy (OIT) is a promising therapeutic approach for children with persistent IgE‐mediated cow's milk allergy (CMA). The prevalence of life‐threatening anaphylaxis in children with persistent CMA undergoing OIT is nearly two times lower (3.5% vs. 6.3%) than in those who stop the protocol. Patients who stop OIT present more frequently severe reactions, requiring intensive care unit admission, or fatal.

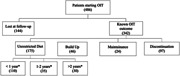

## 1 INTRODUCTION

Cow's milk is one of the most common allergens involved in IgE‐mediated food allergies in children.^1^ Cow's milk allergy (CMA) may cause severe allergic reactions and anaphylaxis, but it carries a good prognosis with a high rate of spontaneous resolution and tolerance acquired by the age of 5 in more than 50% of cases.^2^, ^3^, ^4^ Nevertheless, recent studies have reported an increasing prevalence of persistent CMA,^2^, ^3^ mostly related to the presence of comorbidities as asthma and other food allergies.^5^ Guidelines recommend strict avoidance of the culprit food allergen, which could be challenging, mainly with the most common ones like cow's milk, keeping a high risk of accidental reactions.^6^, ^7^ Furthermore, an exclusion diet may carry nutritional risks, negatively impact the quality of life, and even delay the chances of acquiring immunological tolerance.^6^, ^8^

Oral immunotherapy (OIT) to milk has been considered a promising therapeutic approach for patients with persistent CMA. OIT reduces the risk of reactions after accidental exposure to the allergen, may achieve desensitization, and improves patients' and families' quality of life.^7^, ^9^, ^10^, ^11^ This approach consists of increasing milk doses until reaching the maintenance one.^10^ According to our specific protocol, the first up‐dosing phase is carried out in about 10 days in the hospital, and then milk intake is continued at home.^12^

While being reported as effective in a significant percentage of cases, OIT presents some limits such as adverse reactions, sometimes severe, worsened quality of life due to forced milk intake, and an average 20%–30% failure rate.^13^, ^14^ The principal limit probably concerns the risk of anaphylactic reactions during treatment, which could even be life‐threatening.^11^ Potential risk factors for allergic reactions during OIT have been recently reported, such as older age, a higher degree of sensitization on skin or blood tests, a lower initial reaction threshold, concomitant moderate‐severe asthma, and severe reactions that occurred before OIT.^11^, ^15^ Nonetheless, data on OIT safety remain limited,^2^ and only a few studies in the past decade have evaluated the incidence of adverse reactions during OIT and their risk factors. Furthermore, no study evaluated the risk of severe reaction in subjects who abandoned OIT.

For this reason, we specifically reviewed the rate of severe reactions in our historical cohort of patients who underwent OIT, including those who abandoned the protocol.

## 2 METHODS

This retrospective cohort study was performed at the Allergy Unit of the Pediatric Department, Institute for Maternal and Child Health IRCCS Burlo Garofolo, Trieste, Italy, among a cohort of children affected by persistent CMA who started OIT from February 2000 to January 2020. Patients aged 5–17 years were enrolled at the time of OIT induction, and data from patients whose desensitization status was known by April 2021 were analyzed.

The study was approved by Burlo Garofolo Ethical Committee, Institutional Review Board RC 34/18. Written informed parental consent was obtained for both OIT and data collection.

## 3 OIT PROTOCOL

The OIT protocol consisted of escalation, build‐up, and maintenance phases. The OIT process started at the hospital for the first phase of rapid increase (escalation) in milk dose, following the Specific Oral Tolerance Induction (SOTI) protocol, as already described.^12^ During this phase, children were offered increasing doses of milk, at first diluted in water and then whole. The solution concentration was increased periodically to reach the total milk dose gradually (Table S1). After this, the build‐up phase was conducted at home: patients were instructed to follow a specific increasing protocol adapted to each child outcome after the hospital phase.^16^ Discharged patients with a tolerance higher than 495 mg were required to first increase by 66 mg every 2 or 3 days to 1.98 g, and then by 165 mg every 2 or 3 days up to a single maximum dose of 8.25 g. Patients discharged with less than 495 mg, and more than 165 mg had to increase the dose by 33 mg every 5–7 days up to 990 mg and continue as already described above. Subjects discharged with a dose of less than 165 mg were advised to increase by 16.5 mg every 7–10 days (Table S2). The first CM dose at home was always 66 mg less than the last hospital quantity to reduce the risk of reactions of the first dosage at home. A tailor‐made written emergency treatment plan for the weight was discussed with the parents and delivered to all children. Parents were provided with a dedicated telephone number available 24 h a day for consultations and emergencies. Parents were asked to avoid children's physical exercise after 2 h from the milk intake and to empirically halve the dose during febrile infections or tooth loss. In all cases, dose increases were flexible and could be modified according to each child's reactivity. The sublingual OIT, which consisted of keeping 66 mg of CM under the tongue for 3 min and then spitting it out, was recommended to patients discharged with a tolerance of less than 66 mg of CM. Finally, the third phase of the protocol (maintenance) was conducted at home, and it consisted of the daily assumption of the highest tolerated CM dose, as reached in the previous phase.

The follow‐up program was personalized, and visits were scheduled based on the grade of tolerance reached and adverse reactions.

Ig‐E levels for whole milk, casein, β‐lactoglobulin, and α‐lactalbumin, as well as specific IgG4, were registered at admission and at follow‐up when performed, as well as the presence of other food allergies (none, one, or more than one), celiac disease, or asthma. According to our protocol, all patients requiring IM epinephrine for adverse reactions were instructed by protocol to activate emergency services immediately.

## 4 ADVERSE EVENTS DEFINITION

Parents were asked to report and register adverse reactions during the whole period of OIT. Anaphylactic and nonanaphylactic reactions were defined according to the World Allergy Organization Anaphylaxis Guidance 2020 definition.^17^, ^18^ Nonanaphylactic reactions were arbitrarily defined as the presence of single organ involvement with cutaneous (urticaria, pruritus, lips itching, and angioedema), upper respiratory (nasal symptoms, itchy throat, and cough without bronchospasm), and conjunctival (erythema, pruritus, and tearing) symptoms. Anaphylactic reactions were classified as those that involved two or more organs. Mild anaphylactic reactions were defined as the presence of lower airway (mild bronchospasm) and gastrointestinal (diarrhea, vomiting, and abdominal cramps) symptoms. Moderate reactions have been described as the presence of lower (severe bronchospasm) or upper (laryngeal edema with stridor) airway symptoms. Finally, severe/life‐threatening reactions were arbitrarily defined as the onset of respiratory symptoms and/or collapse/hypotension and/or loss of consciousness requiring the use of IM epinephrine and ED, ward, or PICU admission.

## 5 INCLUSION AND EXCLUSION CRITERIA

The inclusion criteria were arbitrarily defined as age over 5 years (to reduce the risk of including children with a higher incidence of developing spontaneous tolerance), the first OIT attempt, and adequate parental consent.

Children younger than 5 years and older than 17 years, with a previous history of OIT to milk or any other food or requiring treatment with Omalizumab were excluded.

Although asthma was not considered an exclusion criterion, by protocol, all patients included in this study only started OIT after satisfactory asthma control was achieved.

## 6 DATA COLLECTION

Parents were contacted by phone, email, or during the control visits. They were asked the number of adverse reactions at home (0–5, 5–10, and >10), the characteristics and time of the most serious ones (in the first year of OIT, subsequent, or equally distributed), the maximum milk dose tolerated at home, the need and time of adrenaline administration, the hospitalization for adverse reactions, the diet managed at home (free diet, persistent OIT, maintenance phase, discontinuation of treatment due to severe adverse reactions, or development of food aversion), and the time required to achieve an unrestricted diet. To those patients who needed hospitalization for severe adverse reactions, we asked the age at that time, the therapy administered before, during, and after admission, the need for epinephrine administration at home, the hospitalization length, the necessity for either intensive care or intubation, and the outcome after discharge (OIT interruption or maintenance).

The study's primary aim was to analyze the prevalence of life‐threatening anaphylaxis in children with persistent CMA undergoing OIT. The secondary objective was to evaluate potential risk factors for life‐threatening reactions.

## 7 STATISTICS

Descriptive statistics were employed to characterize clinical features, demographics, and comorbidities, while nondescriptive statistics were used to study the link between severe anaphylactic reactions and the considered variables. Fisher's exact test and Wilcoxon–Mann–Whitney test were utilized to analyze the correlation between categorical and continuous variables, respectively. Each result was considered significant if *p* value was <0.05.

## 8 RESULTS

We considered 486 patients who underwent OIT for CM from 2000 to 2020 at our hospital. Of these subjects, 81 could not be contacted due to errors in their data registration (changed or incorrect telephone number), and 63 did not answer the phone after at least five different contact attempts for 2 months. We contacted 342 patients aged 6–26 years by phone, and all of them agreed to participate in the survey. Their median (interquartile range [IQR]) value of the reactivity threshold in the hospital was 231 (66–528) mg. Two hundred sixty‐six (77.8%) had one or more multisystemic reactions before starting OIT, and 105 (30.7%) had a multisystemic reaction the year before the beginning of the therapy.

Cow's milk protein‐specific IgE levels were arbitrarily divided into six severity groups: Class 1 (very low, 0.35–0.69 kU/L), Class 2 (low, 0.70–3.49 kU/L), Class 3 (moderate, 3.50–17.49 kU/L), Class 4 (high, 17.50–52.49 kU/L), Class 5 (very high, 52.50–99.99 kU/L), and Class 6 (abnormal, ≥100 kU/L).

Patients' characteristics are described in Table 1.

**Table 1:** Population characteristics

Characteristics	*n* (%)			
**Male**	219 (64.0)			
**Female**	123 (36.0)			
**Median IQR age (years)**	6.5 (5–10)			
**Median IQR reactivity threshold (ml)**	7 (2–16)			
**Previous systemic reactions**				
≥1	266 (77.8)			
1 in the previous year	105 (30.7)			
**Asthma**	141 (41.2)			
**Other food allergies**	203 (59.4)			
**IgE classes**	**Cow milk**	**α‐lactalbumin**	**β‐lactoglobulin**	**Casein**
1	3 (0.9)	3 (0.9)	5 (1.6)	2 (1.5)
2	15 (4.4)	23 (6.7)	44 (14.4)	20 (6.4)
3	53 (15.5)	69 (20.2)	85 (27.8)	65 (20.8)
4	67 (19.6)	80 (23.4)	90 (29.4)	51 (16.4)
5	87 (25.4)	59 (17.2)	40 (13.0)	52 (16.7)
6	113 (33.0)	73 (21.3)	32 (10.5)	117 (3.5)

Abbreviation: IQR, interquartile range.

Table 2 shows the number and type of adverse reactions during the second phase of OIT carried out at home, from the beginning to the end of OIT (either for withdrawal or successful outcomes). These adverse reactions were homogeneously distributed over the second phase in 188 (55.0%) patients, while 146 (42.7%) presented adverse reactions mainly in the build‐up period and 8 (2.3%) in the maintenance one.

**Table 2:** Number and type of adverse reactions during the second phase of OIT

	*N* (%)
**Number of reactions**	
0–5	142 (41.5)
5–10	50 (14.6)
≥10	150 (43.9)
**Type of reaction**	
None	44 (12.8)
Oculorhinitis	13 (3.8)
Urticaria	108 (31.6)
Asthma or other respiratory reactions	114 (33.3)
Gastrointestinal symptoms	34 (9.9)
Lipothymia or hyporeactivity	31 (9.1)

Abbreviation: OIT, oral immunotherapy.

**Figure 1:**
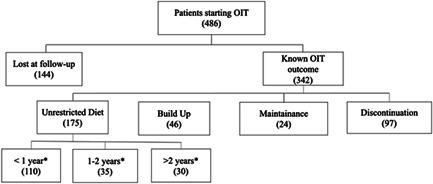
OIT outcomes. *From OIT initiation. OIT, oral immunotherapy

One hundred seventy‐five (51.2%) children reached an unrestricted diet: 110 (32.2%) achieved it in less than 1 year, 35 (10.2%) in 1–2 years, 30 (8.8%) in more than 2 years. Forty‐six (13.4%) are at the build‐up stage. Ninety‐seven (28.4%) stopped the OIT: 67 (19.6%) due to frequent or severe adverse reactions, 23 (6.7%) because of a food aversion, and 7 (2.0%) for other causes not directly linked to the therapy (e.g., interference with daily activities). Finally, 24 (7.0%) children are still at the maintenance stage. The median (IQR) follow‐up time was 9.3 (6.7–11.8) years. Figure 1 shows the OIT outcome of this study.

While assuming milk, 12 patients (3.5%) presented a severe anaphylactic reaction that needed an adrenaline injection. The median (IQR) age was 10 (5–13) years. Eight were managed in the Emergency Department and then discharged at home, while four were admitted to the ward for further treatment or observation. None of them were intubated or admitted to the intensive care unit (ICU). The median (IQR) time between the beginning of OIT and the onset of the life‐threatening event was 283 (77–541) days. Two children who presented a severe reaction immediately abandoned OIT, while the other 10 carried on the treatment. Among the latter, five reached an unrestricted diet with milk and dairy products; three eventually gave up milk intake at a later stage due to frequent and repeated mild reactions; one is still at the build‐up stage (3.96 g of CM protein reached); one remained at the maintenance stage with 1.65 g of CM protein.

Parents of those children who presented severe reactions were asked if there was any known cause: two parents administered the wrong dose of milk, and three recognized further possible triggering elements (e.g., physical activity and contact with other allergens).

Of the 97 children who discontinued the OIT protocol (28.4%) while following a milk avoidance diet, 6 (6.3%) experienced a severe anaphylactic reaction induced by accidental ingestion of cow's milk or dietary products in the subsequent months and years. Two of them died, one was intubated, one was admitted to the ICU, and two were referred to the pediatric ward. For all of them, IM adrenaline was self‐administered out of the hospital. One presented the adverse reaction 1 year after the suspension, one after 7 years, one after 6 years, and two after 8 years. Five out of six had a history of controlled asthma, including the two experiencing a fatal reaction. These two patients did not apparently have additional risk factors. The fatal event was reported to occur as the result of accidental ingestion of milk as a contaminant of other food, and it could not be estimated precisely. Taking into consideration the reactivity threshold they showed during the induction phase of OIT (performed around 6 and 7 years before the fatal event)—respectively, 153 and 272 mg of cow's milk protein—it is likely that they progressively lost the limited and partial tolerance achieved during milk intake. Remarkably they had both stopped OIT because of systemic, but not life threatening, reactions.

Finally, no significant results were found analyzing the link between severe/life‐threatening reactions (either during OIT or after the suspension) and the following variables: age at the beginning of OIT; reactivity threshold; multisystemic reactions before OIT; Ig‐E class for whole milk, casein, β‐lactoglobulin, and α‐lactalbumin; other food allergies; and asthma.

## 9 DISCUSSION

This study showed that the risk of life‐threatening reactions was 3.5% in the group of patients assuming milk during OIT, compared to 6.3% in the group who had discontinued the treatment. Two out of six patients in this latter group experienced a fatal reaction.

The study's primary aim was to evaluate the incidence of severe anaphylactic reactions in patients with CMA who underwent OIT. Of all the patients still assuming milk, 87.2% experienced adverse reactions, but most of them were low‐mild (78.2%), and none were fatal. The risk of dealing with severe reactions and administering intramuscular adrenaline was 3.5%, which dropped to 2% by eliminating possible influencing factors such as incorrect milk dose, contact with other allergens, or concurrent physical activity. Moreover, none of the patients required intensive care. This moderate incidence of risk could be associated with the educational support given to the families about the treatment of eventual adverse reactions, as reported by the EAACI guideline.^18^ Remarkably the group of patients who abandoned OIT showed the worst trend of more severe reactions, even if this difference did not reach statistical significance.

The secondary purpose was to identify the potential risk factors for severe anaphylactic reactions. Other recent studies identified as possible risk factors the older age, the high grade of cutaneous sensitization at prick test, the high IgE level, the lower threshold of initial reactivity, a moderate to severe asthma, and a previous severe reaction before the start of immunotherapy.^11^, ^15^, ^19^ Our study could not demonstrate any statistically significant difference between those patients with severe reactions and the others. This difference between our findings and the other studies may be related to the low rate of severe anaphylaxis that we registered (only 12 out of 344 subjects studied).

Considering the outcome of OIT: 51.2% of patients are actually on an unrestricted diet for milk, 13.4% are in the increasing dose phase, 7% are in the maintaining phase, and 28.4% have suspended the immunotherapy. These data generally agree with those reported by other studies, even if the percentages reported are still different and variegate.^15^, ^20^, ^21^ Overall 71.7% of patients had daily contact with milk, with doses appearing to be high enough to avoid fatal reactions or reaction leading to intubation in case of accidental contact. This event substantially impacted these patients' quality of life, described as significantly improved after the beginning of OIT, despite a lack of prospective assessment through a validated quality of life scale.

Previous studies^15^, ^19^, ^22^, ^23^ evaluated OIT safety, underlying a high incidence of adverse reactions during treatment. Remarkably, as also demonstrated by our findings, most of these reactions were mild, and none were fatal. The percentage of patients who abandoned OIT, mainly for recurrent adverse reactions or the development of a milk aversion, is comparable to other studies (20%–30%).^24^ Regarding the severity of adverse reactions, there is no univocal way to classify them in the literature. We based our retrospective classification on the last EAACI guidelines,^18^ unavailable for most previous studies. Besides, we aimed to give families solid educational support to treat the adverse reactions, as described in our care protocol.^12^ As expected, all studies agreed with the importance of proper educational training for the families to deal promptly and correctly with all adverse reactions.

The strengths of this study are the considerable sample size, higher than the similar previous studies, and the longer time of follow‐up, a mean time of 9.3 years versus approximately 5 years of the previous ones.^15^, ^19^, ^22^ Furthermore, to the best of our knowledge, this is the first study considering the incidence of severe adverse reactions after the OIT suspension. Among the 97 patients who stopped the OIT, 6.3% presented a severe reaction due to possible accidental contact with milk. Two of them died, one was recovered in an ICU, one was monitored for 3 days, and two were referred to the Emergency Care Unit after intramuscular adrenaline self‐administered. This evidence further confirms that an avoidance diet is not 100% safe since milk is strongly present as a contaminant of other food and is difficult to avoid altogether. It could be speculated that when not undergoing OIT, the risk of severe reactions may be higher due to the lack of partial but constant desensitization induced by even small amounts of proteins. Remarkably all children with life‐threatening reactions received intramuscular epinephrine out of the hospital, including the two who presented a fatal outcome, thus confirming that while epinephrine is lifesaving, it is not always foolproof.^13^ Even if the difference in the incidence of anaphylactic reactions between the two populations (ongoing and abandoned OIT) is not statistically significant, the different trends cannot be ignored, particularly in terms of the severity of reactions. While no fatal reactions to milk were reported in all the other studies considering ongoing OIT, the report of such severe episodes after OIT suspension deserves some attention. So far, the data from our study are insufficient to demonstrate a statistically significant correlation between OIT discontinuation and a higher risk of severe reactions, and larger cohorts may be needed to confirm these findings.

Recent studies^25^, ^26^ focused on combined treatment with OIT and biologics—Omalizumab above all—in those patients with a too low reactivity threshold which prevents the beginning of OIT. This strategy seems to be effective in raising the reactivity threshold^14^, ^27^, ^28^ or reducing the number of adverse reactions during OIT. It may be relevant mainly in patients with persistent CM, a previous OIT failure, and with a high recurrence of severe adverse reactions for the accidental contact of milk.^26^ Previous studies addressed the issue of OML effectiveness in children starting their first OIT attempt, showing a better safety record without a significant improvement in the final acquisition tolerance outcome.^29^

In the absence of evident predictive factors of severe anaphylactic reactions for children who undergo OIT, it is still difficult to detect those children who could benefit from Omalizumab administration from the start.

Recently, a limited case series^26^ involving four patients proved the effectiveness of Omalizumab in the setting of a previously failed and abandoned OIT for milk allergy, allowing a safe milk assumption.

If confirmed by further data, this approach could be considered for those patients who prematurely interrupt the OIT due to severe reactions and may be eligible to start treatment with biologics.

This study has some significant limits. First, a significant percentage of patients lost at follow‐up in 20 years. Second, the reactions were not classified in a standardized way due to the retrospective character of the study. Furthermore, the reactions' recording was based on family histories, subjectively experienced and often distant in time. Reactions were graded to reduce this recall bias according to their frequency and the main symptom of the most severe ones. Finally, since this was a single‐center study, the sample size was limited and possibly not big enough to allow statistically significant conclusions.

## 10 CONCLUSIONS

In this study, the risk of life‐threatening reactions was 3.5% in the group of patients assuming milk during OIT, versus 6.3% in the group who stopped the protocol. Although this difference was not statistically significant, the most severe reactions occurred in those children who abandoned OIT, with two of these patients experiencing fatal reactions. Further studies are needed to confirm these data.

Additional studies should also investigate the effectiveness of Omalizumab in the specific setting of OIT failure.

## CONFLICTS OF INTEREST

The authors declare no conflicts of interest.

## ETHICS STATEMENT

This study was approved by the internal review board, with the grant ID RC 34/18.

## AUTHOR CONTRIBUTIONS

Laura Badina and Egidio Barbi conceived the study. Laura Badina, Irene Berti, and Egidio Barbi consistently contributed to the follow‐up of the patients. Sara Babich collected and analyzed data. Francesca Burlo and Beatrice Belluzzi wrote the first draft of the manuscript. Egidio Barbi, Irene Berti, and Laura Badina substantially revised the work. All the authors read and approved the final manuscript.

## Supporting information

Supporting information.

## Data Availability

The data that support the findings of this study are available from the corresponding author upon reasonable request.
