# Supplementary material for: Life‐threatening anaphylaxis in children with cow's milk allergy during oral immunotherapy and after treatment failure
Source: Immun Inflamm Dis. 2022 Mar 29;10(4):e607. doi: 10.1002/iid3.607 (PMC8962636; doi:10.1002/iid3.607)
Supplement: Supplementary file 1 — Supporting information. [file IID3-10-e607-s001.docx]

**SUPPLEMENTARY MATERIAL**

| **Day** | **Dilution (mL of cow’s milk)** | **Dose (mg of cow’s milk protein)** |
| --- | --- | --- |
| 1 | 1 drop^*^ of cow’s milk in 10 mL of water | 0.25 mg, 0.5 mg, 33 mg, 66 mg, 165 mg, 330 mg |
| 2 | 5 drops^*^ of cow’s milk in 20 mL of water | 66 mg, 132 mg, 264 mg, 528 mg |
| 3 | 1 mL of cow’s milk in 20 mL of water | 66 mg, 132 mg, 264 mg, 396 mg |
| 4 | 3 mL of cow’s milk in 20 mL of water | 99 mg, 198 mg, 297 mg, 330 mg |
| 5 | 10 mL of cow’s milk in 20 mL of water | 99 mg, 198 mg, 297 mg |
| 6 | 10 mL of cow’s milk in 10 mL of water | 99 mg, 198 mg, 297 mg |
| 7 | Pure cow’s milk | 66 mg, 132 mg, 198 mg |
| 8 | Pure cow’s milk | 132 mg, 231 mg, 330 mg |
| 9 | Pure cow’s milk | 264 mg, 396 mg, 495 mg |
| 10 | Pure cow’s milk | 429 mg, 528 mg, 660 mg |

**Tab. 1S: Specific Oral Tolerance Induction (SOTI) protocol**

Doses were administered every 1 hour on the first days and every 2 hours on the other days.

^*^1 mL = 20 drops

| **Tolerance dose at discharge (mg of cow’s milk protein)** | **First increase at home (mg of cow’s milk protein)** | **Following increases at home (mg of cow’s milk protein)** | | | |
| --- | --- | --- | --- | --- | --- |
| > 495 mg | 66 mg every 2-3 days up to 1.98 g | 165 mg every 2-3 days up to 8.25 g | | | |
| < 495 mg but > 165 mg | 33 mg every 5-7 days up to 990 mg | 66 mg every 2-3 days up to 1.98 g | | 165 mg every 2-3 days up to 8.25 g | |
| < 165 mg | 16.5 mg every 7-10 days up to 165 mg | 33 mg every 5-7 days up to 990 mg | 66 mg every 2-3 days up to 1.98 g | | 165 mg every 2-3 days up to 8.25 g |

**Tab. 2S: Increasing protocol after the in-hospital phase**

After the in-hospital phase, patients were instructed to follow a specific increasing protocol adapted to their outcome at the discharge.
